# Left Ventricular to Right Atrial Shunt in a Preterm Infant: A Case Report

**DOI:** 10.3390/reports8040238

**Published:** 2025-11-18

**Authors:** Aimann Surak

**Affiliations:** Department of Pediatrics, University of Alberta, 11405 87 Ave NW, Edmonton, AB T6G 1C9, Canada; aimann.surak@ahs.ca

**Keywords:** TnECHO, VSD, preterm

## Abstract

**Background and Clinical Significance:** Premature infants are at high risk of life-threatening complications due to the immaturity of multiple organ systems, and close monitoring in neonatal intensive care units is indispensable for their stabilization, survival, and morbidities. Despite major advances in perinatal care, cardiovascular pathology remains one of the most frequent morbidities in this population. Targeted neonatal echocardiography is implemented in many neonatal intensive care units around the world; it provides earlier and precise assessment during hemodynamic instability, allowing targeted management and follow-up. It can also detect red flags for anatomical heart defects. Left ventricular to right atrial shunt is a rare and presents a challenging diagnostic entity in very premature neonates. **Case Presentation:** We present a case of left ventricular to right atrial shunt in a preterm baby that was diagnosed by the Targeted neonatal echocardiography team. **Conclusions:** The purpose of this case report is to illustrate how targeted neonatal echocardiography enabled the identification of this rare diagnosis in a premature infant, which underscores its growing utility and value in neonatal care.

## 1. Introduction and Clinical Significance

Over the last decade, targeted neonatal echocardiography (TnECHO) has been increasingly used in neonatal intensive care units (NICUs) around the world [1]. In addition to its major role in diagnosing specific hemodynamic pathophysiologies, it is critical in recognizing red flags in abnormal cardiac anatomy [2]. Left ventricular to right atrial shunt (LVRAS) is uncommon in neonates with an anatomically normal heart; however, it has been increasingly recognized and reported [3].

In this report, we present a case of a preterm infant with LVRAS with a co-existing peri-membranous ventricular septal defect (PMVSD). This is the first case of LVRAS to be reported by a TnECHO team.

## 2. Case Presentation

We present a male preterm infant, born at 31 weeks’ gestation to a 33-year-old primigravida mother. Pregnancy was complicated by premature ruptured membranes, gestational diabetes, and placental abruption. The infant was delivered by cesarean section, indicated by breech presentation and fetal heart rate decelerations. Deferred cord clamping was not performed due to concerns about placental abruption. Resuscitation included positive-pressure ventilation, followed by a quick transition to non-invasive continuous positive airway pressure (CPAP). APGARs were 7 and 9 at 1 and 5 min, respectively. Birth weight was 1600 g. The infant was admitted to the NICU for ongoing care and management. A central umbilical venous catheter was inserted successfully.

Initially, the infant remained on CPAP with minimal oxygen and was trialed off on day 5 of life, which he tolerated well. A loading dose of caffeine was given after birth, and a daily maintenance dose continued. Trophic feeds were initiated shortly after delivery, and feeds progressed gradually. The infant remained in room air, with no increased work of breathing. Subsequently, intravenous parenteral nutrition was weaned off, and full feeding was achieved on day 10 of life.

**Figure 1:**
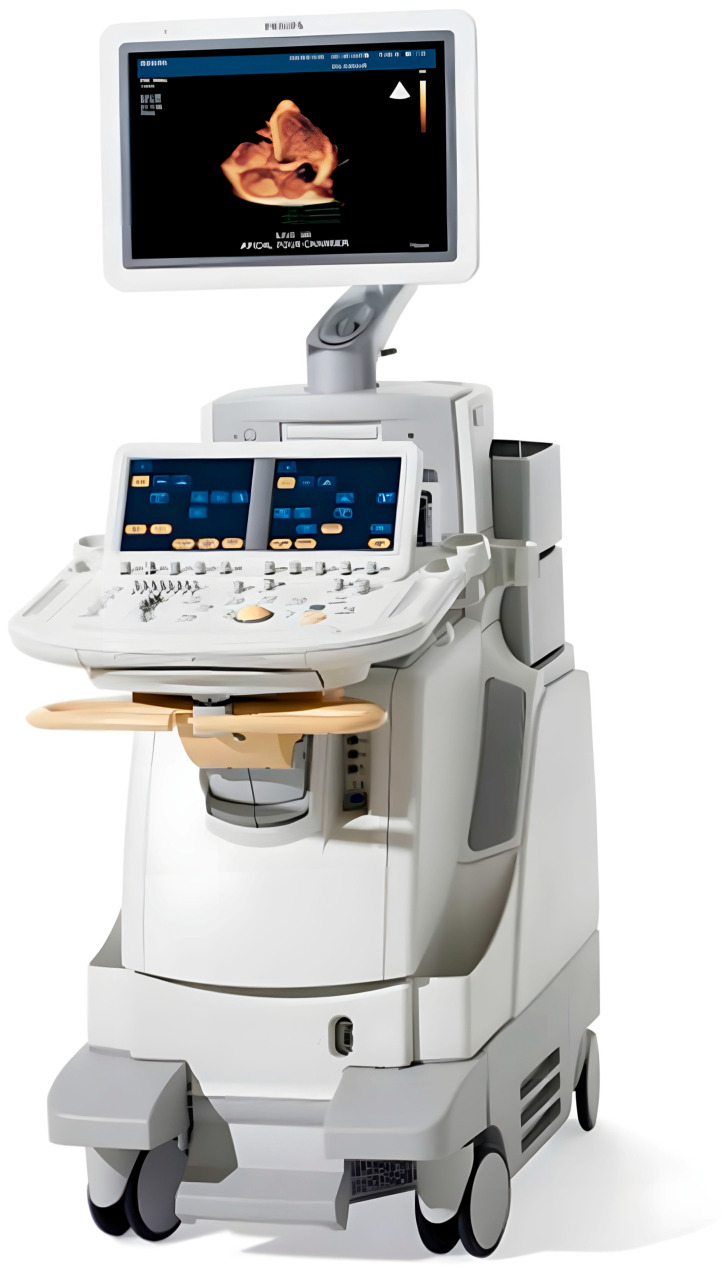
TnECHO machine used for diagnosis.

**Figure 2:**
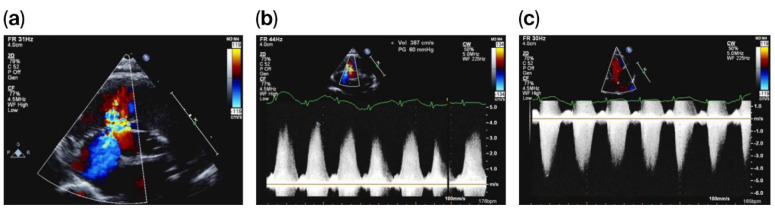
PMVSD with adjacent eccentric tricuspid regurgitation (**a**), with VSD Doppler (**b**) and tricuspid regurgitation Doppler (**c**).

On day 20 of life, and due to a heart murmur on auscultation, TnECHO was requested. At our center, we perform TnECHO using a Philips iE33 Ultrasound Machine with a high-frequency 12 MHz phased-array probe (Figure 1). Echocardiography findings revealed a large peri-membranous ventricular septal defect (PMVSD) measuring approximately 4 mm, with left-to-right restrictive flow, with a peak gradient of 4 m/s, as shown in Figure 2. Biventricular systolic function was normal. Echocardiographic markers of pulmonary hypertension were reassuring and revealed a rounded intraventricular septum during systole (systolic left ventricular eccentricity index of 1), demonstrating a parabolic mean pulmonary artery flow Doppler pattern, with a pulmonary artery acceleration time to right ventricular ejection time (PAAT/RVET) ratio of 0.35 and a right ventricular output of ~400 cc/k/min. Tricuspid annular plane systolic excursion (TAPSE) was 8.5 mm, right ventricular fraction area change (RVFAC) was 40%, and tricuspid valve S’ (TV S’) was 5 cm/s. However, there was a significant tricuspid regurgitation jet with a peak velocity of 4 m/s, estimating a right ventricular peak systolic pressure of 70 mmHg, which is at the systemic level. Of note, this regurgitation jet was eccentric, adjacent to the VSD jet, as shown in Figure 2.

In consultation with pediatric cardiology, the diagnosis of pulmonary hypertension was deemed unlikely. This was due to the reassuring echocardiographic markers (other than tricuspid regurgitation) and clinically stable status. Chest X-ray was also reassuring and negative for pulmonary edema. A diagnosis of LVRAS was suspected by the pediatric cardiology team as an etiology for the tricuspid regurgitation jet. Conservative management was recommended. The infant remained in room air and hemodynamically stable. Subsequently, the infant was discharged home at approximately 36 weeks of corrected gestation. The infant was followed up as an outpatient at the local pediatric cardiology clinic; the tricuspid regurgitation was not observed and was likely to have subsequently resolved. The infant has been growing well with no clinical concerns.

## 3. Discussion

LVRAS was first described in 1958 by Gerbode et al. [4]. It can be either congenital (Gerbode defect) or acquired [5,6,7,8]. Normally, the tricuspid valve is located below the mitral valve. Hence, a part of the septum between these two chambers might become ruptured as a complication of cardiac surgery, endocarditis, trauma or myocardial infarction [9].

**Figure 3:**
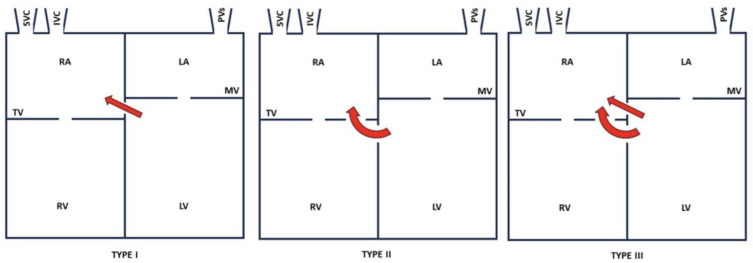
Types of LVRAS. IVC: inferior vena cava; SVC: superior vena cava; PVs: pulmonary veins; RA: right atrium; RV: right ventricle; TV: tricuspid valve; LA: left atrium; LV: left ventricle; MV: mitral valve.

Anatomically, this may be above (type I), or below the tricuspid valve (type II) or both (type III), as shown in Figure 3 [4,10]. In the latter two cases, a PMVSD often exists in the tricuspid valve septal leaflet. The tricuspid valve may be abnormal (cleft, widened commissural space, or perforated) [11,12]. An abnormal case of tricuspid chordae causing regurgitation has also been reported [13].

The diagnosis of LVRAS is challenging due to the diagnostic accuracy of echocardiography. Therefore, it is important to always consider LVRAS when an unexplained turbulent flow is observed in the right cardiac chamber. Other modalities used for diagnosis include transesophageal echocardiography (TEE), cardiac magnetic resonance imaging (CMRI), and computed tomographic angiography [14]. As TnECHO is often the first modality used in many NICUs, it has become a crucial first step in the diagnosis (screening) of congenital heart defects, highlighting its diagnostic value and the prognostic implications.

In our case, the echocardiography findings were likely consistent with type II LVRAS with a co-existing PMVSD similar to what is described in the literature. Our case confirms the importance of integrating echocardiographic markers with components of a clinical exam to establish the diagnosis. Additionally, our TnECHO team recognized multiple other defects, providing an early diagnosis of a significant cardiac condition and allowing for earlier intervention. Guidelines on the use of TnECHO have been adopted to optimize monitoring in the NICU [15,16,17]. Additionally, in our case, the assessment occurred at day 20 of life due to the clinical concerns mentioned above; it is possible that an earlier TnECHO would have resulted in an earlier diagnosis. As the infant remained stable during the NICU course, an earlier assessment would probably not have resulted in a better outcome in this particular case. However, in other cases of pathological heart defects, an earlier diagnosis may impact the outcomes.

Implementation of this tool is associated with improved outcomes, refs. [18,19] and it facilitates “precision medicine” by detecting pathophysiological factors and continuously evaluating fluctuations in hemodynamics [20]. In conclusion, our case is a good example of the successful use of TnECHO in diagnosis, management, and follow-up in the NICU. Additionally, high suspicions of red flags for heart defects while performing TnECHO should always be followed; furthermore, collaboration and ongoing discussions with the pediatric cardiology team are also important.

## 4. Conclusions

TnECHO can detect red flags for rare congenital heart disease. Careful evaluation of every murmur is crucial, and TnECHO is beneficial in this regard.

## Data Availability

The original data presented in the study are included in the article, further inquiries can be directed to the corresponding author.
